# Locals

**Published:** 1879-04-20

**Authors:** 


### *LOCALS.*

THE BEDFORD IRON AND ALLUM SPRINGS.—The efficiency of these waters, and the mass prepared from them, is now generally conceded. Those desiring to see a reliable account of the properties of these remarkable waters from a responsible and intelligent source, should read the article in present copy of our Journal, by Prof. J. J. Moorman, which we copy from a Maryland Journal. Being satisfied from certain cases in our own practice, and from the testimony of others, that the iron and alum mass is a very valuable therapeutic agent, we trust that our readers will test it in practice, and report results to this Journal. See advertisement on this subject in present number.

THE BUFFALO LITHIA WATERS.—It is common with a certain class of medical men to claim that the vegetable kingdom cotains a specific for all the ills that flesh is heir to; yet in a certain class of affections, as for instance *the diseases peculiar to women*, it is believed that no vegetable production has been found to equal the above waters. They meet also other important indications. See advertisement on first colored page of this Journal.

